# Coconut Shell Carbon Preparation for Rhodamine B Adsorption and Mechanism Study

**DOI:** 10.3390/molecules29174262

**Published:** 2024-09-09

**Authors:** Jinrui Yu, Yifan Bian, Rongfeng Wang, Shiping Zhou, Zhongying Wang, Dawei Wang, Huijuan Li

**Affiliations:** Key Laboratory of Efficient Utilization of Forest Biomass Resources in Southwest China, National Forestry and Grassland Administration, Southwest Forestry University, Kunming 650224, China; m13610717421@hotmail.com (J.Y.); yiguangshi5@gmail.com (Y.B.); w_rong_feng@163.com (R.W.); kmzhoushiping@163.com (S.Z.); 18387551371@139.com (Z.W.); wdwchem@163.com (D.W.)

**Keywords:** coconut shell carbon, rhodamine B, adsorption, adsorption mechanism

## Abstract

Phosphoric acid is used as a chemical activator to prepare coconut shell carbon (PCSC), and for investigating rhodamine B (RhB) adsorption performance. The optimal conditions for the preparation of PCSC (calcined temperature, phosphoric acid concentration), and the influence of adsorption conditions (concentration, pH, etc.) on RhB and the recovery performance of optimal carbon are investigated. Experimental results show that when the amount of PCSC (600 °C, 2 h) is 0.2 g, the initial RhB concentration is 10 mg/L, pH = 6, and the adsorption time is 30 min, it can have 95.84% RhB adsorption efficiency. Liquid ultraviolet spectroscopy also supports this adsorption performance. Characterization data showed that hydroxyl and ester groups, aromatic structures, and PO_4_^3−^ existed on the surface of PCSC, and the amount decreased with increasing calcined temperature. PCSC has a BET (N_2_) surface area of 408.59 m^2^/g and has a micropore distribution, EDS-detected P content is 3.91%. SEM showed that the PCSC formed micropores which could better adsorb RhB. The kinetic and thermodynamic analysis of the adsorption of RhB by PCSC showed that the adsorption process was in accord with quasi-secondary kinetic equations and ΔG^θ^ was between −1.65 and −18.75 kJ/mol. The adsorption was a physical adsorption and a spontaneous endothermic reaction, and the obtained PCSC sorption isotherms were classified as Langmuir-type. The RhB adsorption mechanism on PCSC includes pore diffusion, hydrogen bonding, and π−π conjugation. The PCSC prepared by H_3_PO_4_ modification has superior adsorption and recycling performance for RhB, providing a reference for the preparation of other biomass carbon materials for the treatment of dye wastewater.

## 1. Introduction

Wastewater commonly includes pharmaceutical, pesticide, dye, and heavy metal ion waste. Among these, dye wastewater has high chromaticity and contains large organic molecules, which are difficult to biodegrade. Discharge of dye wastewater into natural water can affect the self-purification ability of the water and endanger the survival of aquatic organisms. Therefore, efficient treatment of dye wastewater is of great significance for environmental governance and the protection of the ecological environment [1,2]. Commonly used methods for the removal of RhB and other dyes include physicochemical, chemical and biological methods, such as coagulation and precipitation [3], membrane separation [4], adsorption [5], chemical oxidation [6], ion exchange [7] and aerobic and anaerobic microbial degradation [8]. Among the above methods, the adsorption method is considered to be an effective method to treat dye wastewater. The use of agricultural and forestry waste as a carbon source to prepare biochar as an adsorbent has the advantages of wide availability of raw materials, low cost, and high pollutant removal efficiency, which has attracted widespread attention. Biochar is a kind of highly aromatic, carbon-rich porous solid particulate, which is generated by pyrolysis of carbon-rich biomass under anaerobic or anoxic conditions [9]. It contains a large amount of carbon, and has a large surface area and various kinds of oxygen-containing active groups on the surface, and also has a rich pore structure. It has been reported that biochar is used to adsorb dyes and other pollutants in water environments [10]. Zhe Sun et al. [11] prepared a bimetallic MOF anchored corncob calcined derived activated carbon (CCAC) by a one-step solvothermal method. The obtained porous carbon provided a high specific surface area for stable MOF support and served as an organic pollutant buffer reservoir, which was advantageous for efficient photocatalytic degradation of organic pollutants. The optimized MOF/CCAC-5 samples had a 100% degradation rate for RhB under visible light. Charred Irvingia gabonensis endocarp waste (DNc) was used and coated with chitosan (CCDNc). Inyinbor, A. A. et al. [12] prepared RhB adsorbents. The maximum monolayer adsorption capacities obtained from the Langmuir equation are 52.90 and 217.39 mg/g for DNc and CCDNc, respectively.

Following the vast applications of AC materials, it is interesting to explore new sources of low cost, easily available, carbon rich and low ash precursors with desired physicochemical properties [13]. Since the 1980s, the total production of coconut in the world has reached more than 5 million tons, resulting in a large amount of coconut husk waste. Coconut shell biomass activated carbon has the characteristics of large surface area, well-developed pores, rich functional groups and low price, and is beneficial for use as adsorbents [14]. Using coconut spathe (CS) and KOH as an activating agent under nitrogen atmosphere via the pyrolysis method, Prashanthakumar, T.K.M., prepared highly porous carbon material. KCS (800 °C) reached maximum BET surface area (1705 m^2^/g) and maximum pore radius (2 nm). The total acidic sites were 40.1 mmol/g and basic sites were 18.62 mmol/g. It also had 275 mg/g 4-CP adsorption performance [15]. Kodali Jagadeesh [16] prepared activated coconut charcoal as a super adsorbent for organophosphorous pesticide removal. The adsorption capacity reached 228.1 mg/g and 258.6 mg/g. Ayodele Rotimi Ipeaiyeda [17] used coconut shell and palm shell as raw materials to study the effects of modification with ammonia and ammonium acetate on the physical and chemical properties, morphology, thermal properties, surface functional groups and pore structure of activated carbon. The surface modification of activated carbon was confirmed by FTIR spectroscopy. Currently, there is relatively little research on the adsorption of low concentration RhB by coconut shell carbon, and there are few literature reports on its adsorption mechanism on RhB.

Considering the above references, in this paper, the biomass coconut shell (CS) modified by H_3_PO_4_ was used to prepare porous carbon materials for RhB removal. It focuses on the preparation of activated carbon from coconut shells and explores the effects of preparation methods, adsorption temperature, initial concentration, solution pH, recovery performance and other conditions on the adsorption of RhB by PCSC. At the same time, the thermodynamic and kinetic properties of the adsorption reaction of RhB by PCSC are investigated, providing new ideas for the resource utilization of coconut shells.

## 2. Results and Discussion

### 2.1. PCSC Adsorption Ability

#### 2.1.1. Effects of Treated Phosphoric Acid Concentrations Impregnation

**Figure 1:**
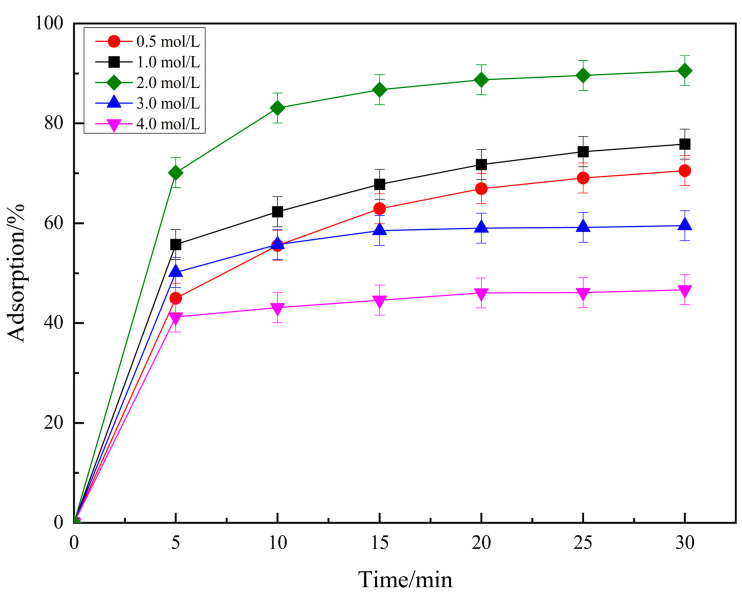
Effects of impregnation of phosphoric acid at different concentrations.

The Effect of phosphoric acid concentration on carbon adsorption performance is shown in Figure 1. When the H_3_PO_4_ concentration increases to a certain range, the adsorption capacity of PCSC on RhB also increased. When the concentration of H_3_PO_4_ reached 2.0 mol/L, the adsorption rate reached 90.53%. However, when the concentration of phosphoric acid is greater than 2 mol/L, the adsorption performance of PCSC decreased. Therefore, 2.0 mol/L H_3_PO_4_ was selected as the activator concentration.

#### 2.1.2. Effect of PCSC Activation Temperatures by H_3_PO_4_ on RhB Adsorption

**Figure 2:**
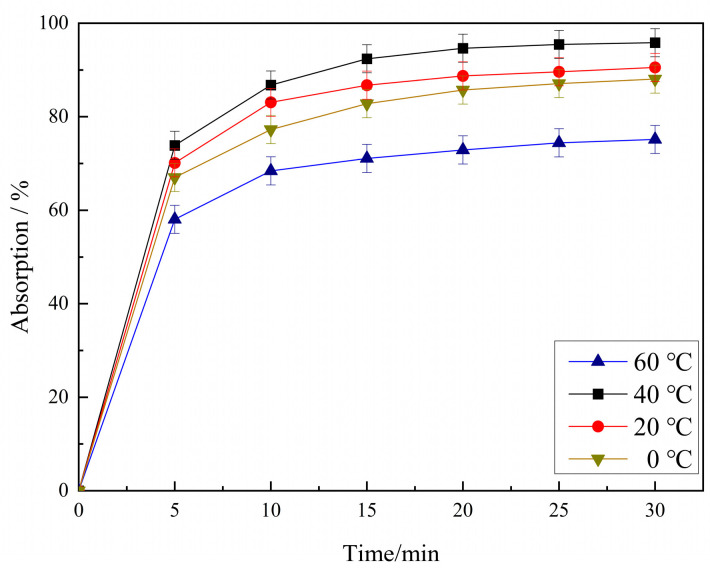
Effects of H_3_PO_4_ impregnation temperatures on RhB removal.

Figure 2 shows the adsorption experiment of 10 mg/L RhB on PCSC, which was impregnated in a 2.0 mol/L phosphoric acid water bath at 0 °C, 20 °C, 40 °C, and 60 °C, and then calcined at 600 °C for 2 h. It can be seen from Figure 2 that the adsorption effect of PCSC on RhB varies with the temperature of the water bath. PCSC activated in a water bath of 2.0 mol/L H_3_PO_4_ at 40 °C has the best adsorption effect on RhB, with an adsorption rate of 95.84%. When the temperature of the water bath was lower than 40 °C, such as 0 °C and 20 °C, it had lower RhB adsorption. When the bath temperature was increased, the adsorption effect was the worst (75.14%). In conclusion, the optimal water bath temperature is 40 °C.

#### 2.1.3. Effect of Calcination Temperature

**Figure 3:**
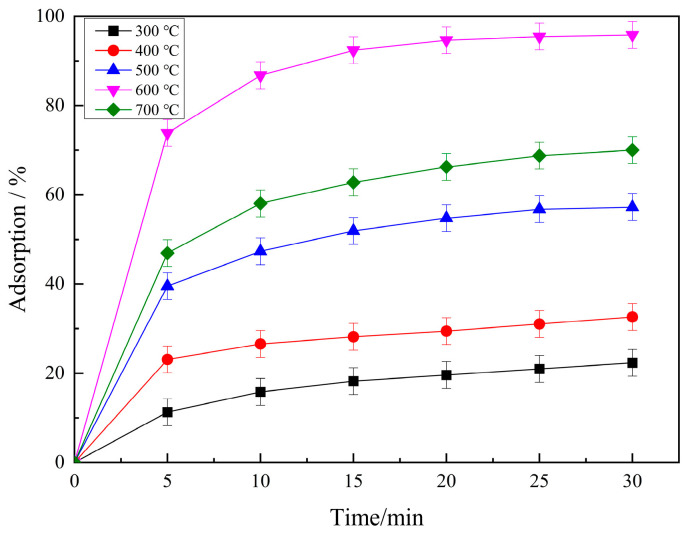
Effects of PCSC calcined temperatures on RhB adsorption.

The calcination temperature affects pore formation, pore size distribution, and surface functional groups, which in turn affects the RhB adsorption performance of the prepared PCSC. As can be seen from Figure 3, the adsorption rate of RhB by PCSC increased first and then decreased with calcined temperature. The PCSC (300 °C, 2 h) has 22.35% RhB adsorption ability, and when calcined at higher temperature, it has greater RhB removement. Among these, PCSC (600 °C, 2 h) had the best adsorption effect on rhodamine B (95.84%), while the adsorption rate at 700 °C was 70.04%. The main component of coconut shell is cellulose. The reaction of H_3_PO_4_ molecules with C proceeds as follows: 4H_3_PO_4_ + 10C → P_4_ + 10CO + 6H_2_O. They could decompose to gaseous H_2_O and CO_2_ to form a pore structure after H_3_PO_4_ activation [18]. When the reaction temperature is less than 200 °C, the carbon material undergoes a high degree of aromatization through phosphoric acid dehydration and catalytic hydroxyl elimination of cellulose. At temperatures greater than 300 °C, and in an anhydrous atmosphere, P_2_O_5_ acts as a Lewis acid and reacts to obtain a large amount of P-O network structure, well-developed micropores, and high specific surface area of the activated carbon. When the temperature exceeds 600 °C, H_3_PO_4_ no longer acts as an activator, and due to thermal shrinkage, the surface area and pore volume of activated carbon decrease, thereby reducing its adsorption performance for RhB [19]. From the FTIR spectra, a peak at 1002 cm^−1^ attributed to P-O vibration decreased, and also large pore distribution appeared in SEM spectra, which is beneficial for RhB adsorption.

#### 2.1.4. The Effect of Different pH

**Figure 4:**
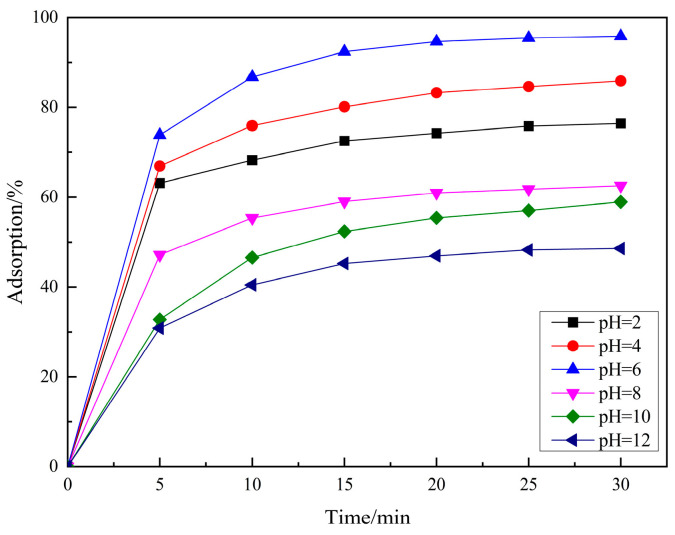
Effects of initial RhB pH on removal for PCSC.

**Figure 5:**
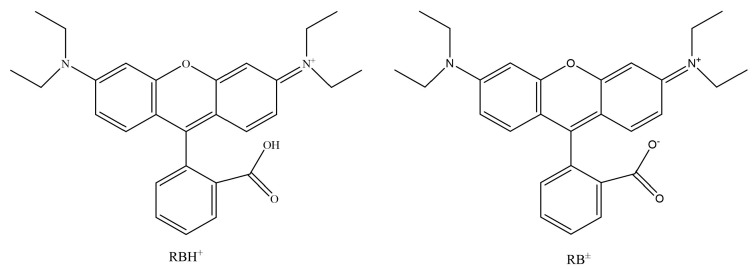
Two forms of RhB.

Figure 4 shows the adsorption experiments of RhB at different initial pH values over PCSC. The solution was adjusted with acetic acid and ammonia in order to obtain its acidic and basic solution. It can be seen that when the pH of the solution is 2, 4, 6, 8 and 10, the adsorption rates of PCSC is 76.43%, 85.93%, 95.84%, 62.57%, 58.93% and 48.57%, respectively, which shows that PCSC adsorption performance in acidic and neutral solutions is better, but in alkaline conditions is worse. The pH of the solution can affect the form of RhB (shown in Figure 5) and the surface charge of PCSC. When the pH is low, RhB molecules exist in the form of RBH^+^, while when the pH is high, the -COOH in RhB ionizes and exists in the form of RB^±^ zwitterions. Compared to acidic conditions, the adsorption rate is lower under alkaline conditions, which is speculated to be due to the different electrostatic repulsion experienced by RhB when diffusing onto the surface of PCSC, and the surface of PCSC carries negative charges [20]. Under acidic conditions, negative charges are first neutralized, and the surface of PCSC is occupied by positive charges. Similarly, under alkaline conditions, there is a large amount of negative charge on the surface of PCSC, and RhB diffuses towards PCSC with greater repulsion. Therefore, the adsorption effect of RhB molecules is worse under alkaline conditions [21].

#### 2.1.5. Influence of Water Source

**Figure 6:**
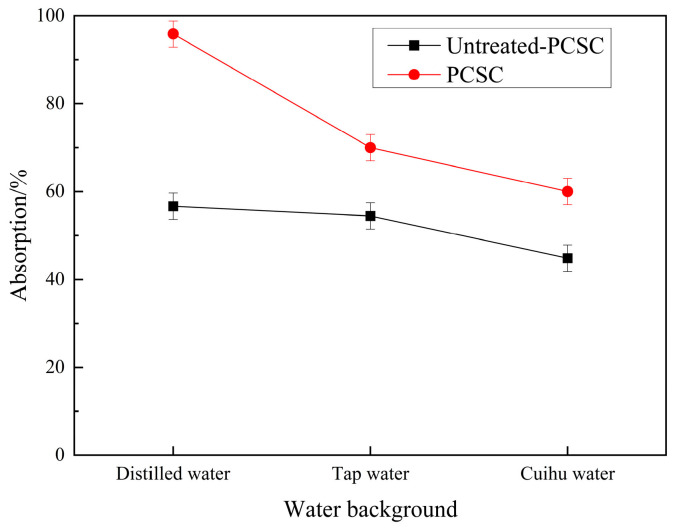
Effects of different water sources on adsorption of RhB.

Figure 6 shows the adsorption experiments of PCSC and untreated PCSC on RhB under different water sources. It can be seen that the optimal carbon has a better adsorption effect on RhB under different water sources than untreated PCSC. The possible reasons are that the activated coconut shell carbon with H_3_PO_4_ is rich in micropores, and contains PO_4_^3−^, and secondly, that the adsorption effect of coconut shell carbon on RhB in distilled water is better than that of tap water, and tap water is better than that of Cuihu water. The reason may be that there is a large amount of salt and algae in tap water and Cuihu water, and these have competitive adsorption with RhB, thus decreasing the PCSC adsorption performance for RhB.

#### 2.1.6. The Degradation Effect of Different Pollutants

**Figure 7:**
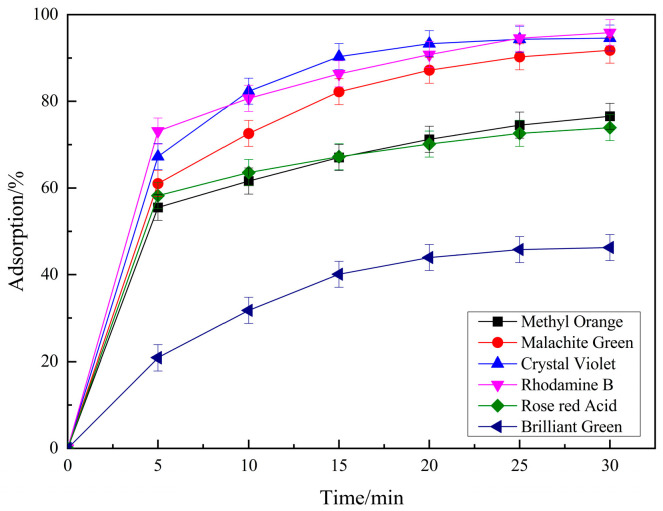
The degradation effect of different pollutants.

Methyl orange, malachite green, crystal violet, bright green and rose red acid were used for considering the application of prepared PSPC. The results are shown in Figure 7. It can be seen that the prepared PSPC has a certain adsorption effect on several selected dyes. Among them, except for RhB, it can achieve an adsorption performance of over 90% for crystal violet and malachite green, about 76% removal effect for rose red acid and methyl orange, and 46.26% adsorption performance for bright green, reflecting the excellent adsorption characteristics of the prepared PSPC on organic dyes. Table 1 shows the relationship between dye properties and adsorption rates. It can be seen that the cationic and anionic characteristics of dyes have a significant impact on the adsorption performance of PCSC. Under the same conditions, cationic dyes are more favorable for adsorption on PCSC, while anionic dyes are relatively poor. Mainly due to the negative charge on the surface of PCSC, it has a repulsive effect with anionic dyes, resulting in a decrease in adsorption performance.

#### 2.1.7. Cyclic Adsorption Performance of PCSC

**Figure 8:**
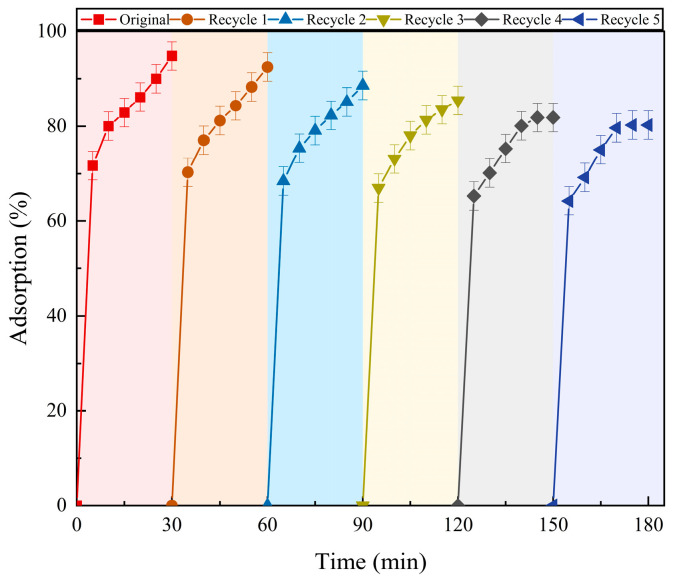
Cyclic adsorption performance of PCSC.

PCSC was used to adsorb 10 mg/L RhB at a dosage of 2 g/L. After 30 min of adsorption, the material was washed with 99.5% ethanol, dried, and then subjected to adsorption again. This process was repeated for a total of 6 cycles to test its cyclic adsorption performance. The cyclic adsorption performance of PCSC is shown in Figure 8. The maximum adsorption efficiency of PCSC gradually decreases with increasing cycle numbers. However, after 6 cycles, the adsorption efficiency of PCSC still remains above 85.25%, indicating that PCSC has good cyclic recovery capability and long-term stability.

#### 2.1.8. Effect of Biochar Species Activated by H_3_PO_4_ on RhB Adsorption Efficiency

**Figure 9:**
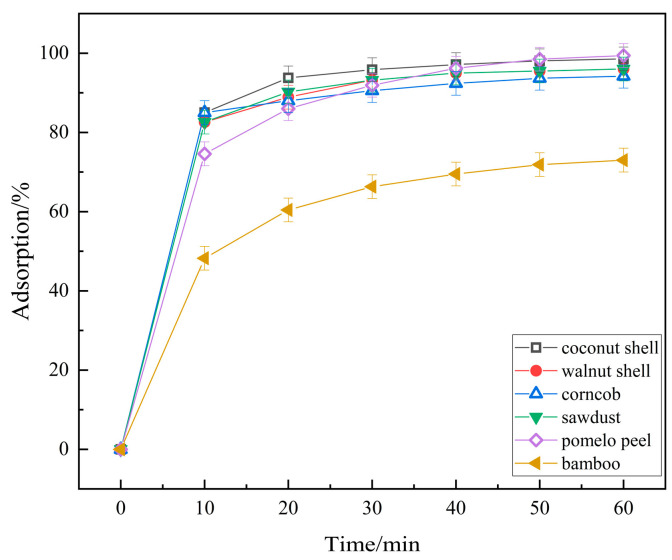
Effect of Biochar species activated by H_3_PO_4_ on RhB Adsorption efficiency.

Under the same H_3_PO_4_ treatment concentration (2 mol/L) and at a water bath temperature of 40 °C, several common agricultural and forestry wastes (coconut shells, walnut shells, corn cobs, sawdust, pomelo peels, and bamboo) were pretreated for 4 h to prepare biochar. Using the same characterization method, 0.2 g biochar was added to 10 mg/L, 100 mL RhB in an adsorption experiment. The results are shown in Figure 9. As indicated by Figure 9, among the different biomass chars treated with H_3_PO_4_, all materials exhibited adsorption performances ranging from 94.26% to 99.48% after 60 min, except for bamboo charcoal, which showed an adsorption performance of only 73.03%. The adsorption performances were relatively consistent. This suggests that the biomass chars prepared through H_3_PO_4_ modification in this study possess a generally applicable capability for adsorbing RhB contaminants.

#### 2.1.9. Comparison with Other Adsorbents

Currently, several materials reported in the literature focus on RhB removal. Some of the other reported adsorbents are listed in Table 2. Compared with these, PCSC had a near neutral environment and a significant advantage in RhB adsorption capacity, which also indicates the potential of RhB adsorbent materials.

#### 2.1.10. Liquid UV Characterization of PCSC

**Figure 10:**
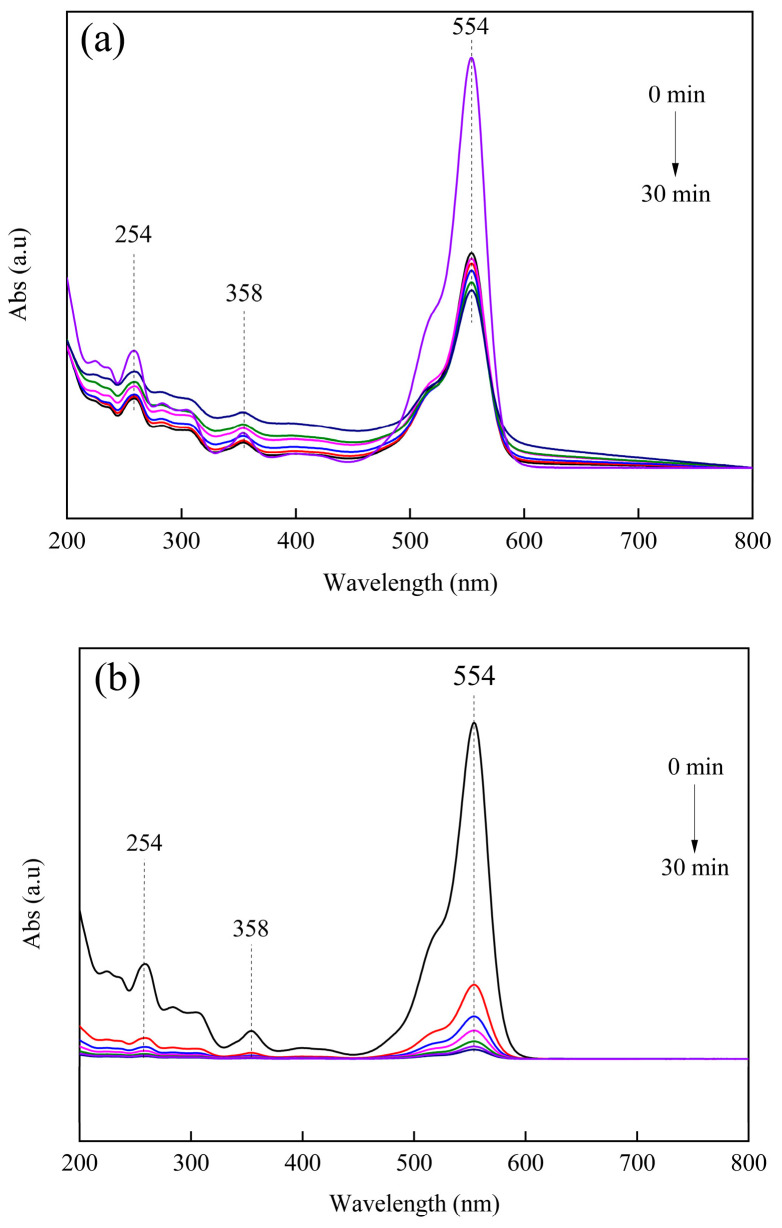
(**a**) UV-VIS adsorption of RhB on untreated PCSC. (**b**) Adsorption of RhB on modified PCSC.

The liquid UV spectra of RhB adsorption on untreated PCSC and PCSC are shown in Figure 10a. It can be observed that RhB exhibits a strong vibrational peak at 554 nm, which is caused by the larger conjugated system in RhB. The strong absorption peak at 254 nm and in the range of 250–300 nm correspond to the benzene ring structure in RhB. The absorption peak at 358 nm is C=O, corresponding to the -COOH in RhB. From Figure 10b, it can be seen that the untreated PCSC has poor adsorption efficiency for RhB, with an adsorption rate of only 56.67% at 30 min, and most of the characteristic peaks of RhB still exist. The adsorption rate of PCSC activated by H_3_PO_4_ can reach 95.84% after 30 min. When the adsorption reaction lasts for 10 min, only the absorption peak generated by the conjugated system can be observed, and other peak types almost disappear. However, the characteristic peaks have not shifted, and the skeleton of the conjugated system still exists. Therefore, the adsorption of RhB by PCSC is mainly physical adsorption. PCSC prepared after H_3_PO_4_ activation improves its adsorption capacity for RhB.

### 2.2. PCSC Characterization

#### 2.2.1. BET Surface Area and Pore Structure Analyses

**Figure 11:**
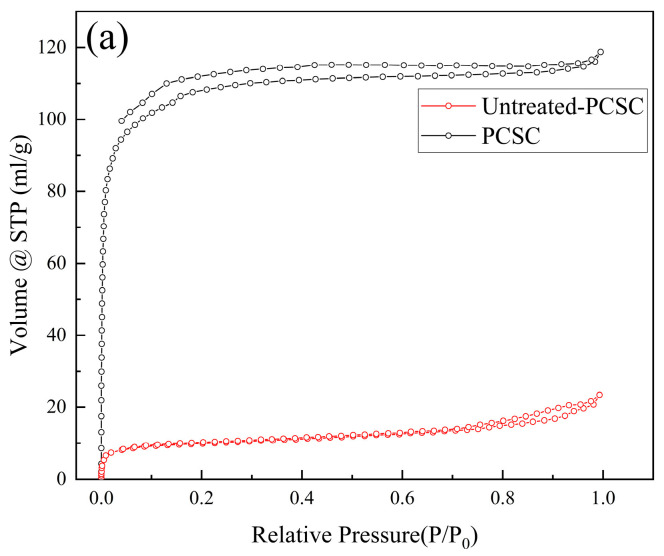

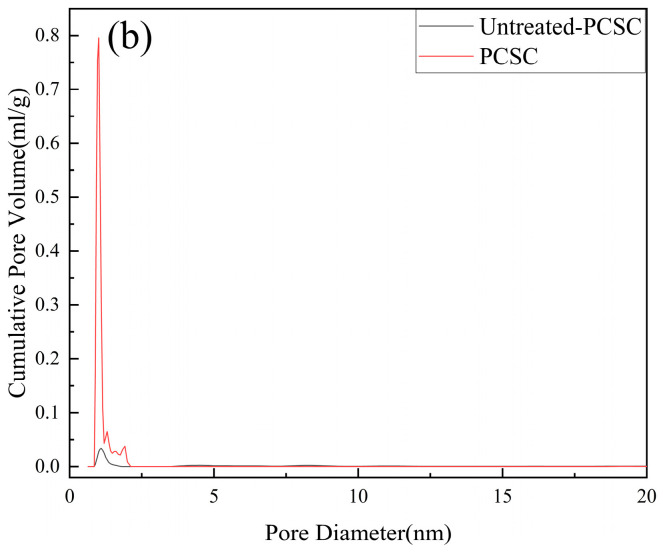
(**a**) N_2_ adsorption/desorption isotherm of PCSC and untreated PCSC. (**b**) Pore size distribution curve of PCSC and untreated PCSC.

In order to investigate the surface area and pore structure of the PCSC and untreated PCSC, the N_2_ adsorption–desorption isotherm and the pore size distribution plot of the two materials are shown in Figure 11a. It can be seen that the isotherm exhibits a typical type I curve, revealing the microporous structure of the sample; meanwhile, the pore size distribution curve in Figure 11b also confirms that the main pore distribution is micropores. Additionally, BET specific surface area of PCSC is 408.59 m^2^/g, which increased to nearly ten times larger than that of untreated PCSC (37.01 m^2^/g). The average pore diameter of PCSC is 1.803 nm, and the untreated is 3.918 nm, suggesting that H_3_PO_4_ treated coconut shell increased the surface area and micropores. Both higher specific surface area and microporous structure could enhance the adsorption capacity of RhB.

#### 2.2.2. FTIR Characterization

**Figure 12:**
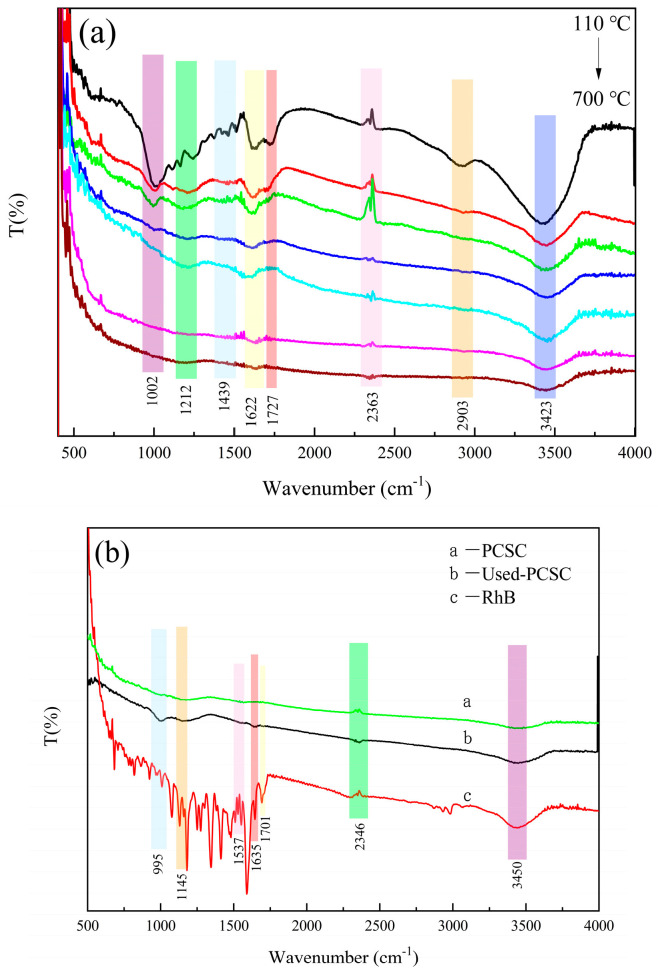
(**a**) FTIR spectra of PCSC calcined at different temperature. (**b**) FTIR spectra of adsorption before and after and untreated PCSC.

Figure 12a shows PCSC calcined at different temperatures after phosphoric acid modification and calcination for 2 h. The infrared spectra of PCSC prepared at different temperatures are shown in Figure 12b, with the stretching vibration peak of P-O at 1002 cm^−1^ and the stretching vibration peak of C-O-C at 1212 cm^−1^. 1439 cm^−1^ is the bending vibration peak of -CH_2_. The stretching vibration peak of C=O is at 1727 cm^−1^, and the asymmetric stretching vibration of CO_2_ is at 2356 cm^−1^, which may be caused by the adsorption of CO_2_ on the sample surface. The surface of PCSC adsorbs water molecules, with bending and stretching vibration peaks of -OH at 1622 cm^−1^ and 3443 cm^−1^, respectively. As the roasting temperature increases, functional groups such as P-O, C=O and C-O-C gradually weaken and disappear, indicating that the increase in roasting temperature promotes the volatilization or carbonization of organic components on the surface of PCSC [26].

The FTIR spectra of RhB, fresh and used PCSC are shown in Figure 12b. H_3_PO_4_ modified PCSC at 1145 cm^−1^ may be caused by P=O stretching vibration, which indicated the formation of P-containing carbonaceous species, which was consistent with the study of Yiping Luo [27]. After adsorption of RhB on PSPC, an absorption band at 3450 cm^−1^, which was the vibration of -OH, becomes stronger, indicating that -OH participated in adsorption reactions. Also, 1537, 1635 and 1701 cm^−1^ appeared after adsorption, which was described as -NH_2_, C=C and C=O, respectively [28], suggesting that the structure of RhB was absorbed on the surface of PSPC. The band located at 995 cm^−1^ was the C-O vibrations of the ether groups. and also band at 1145 cm^−1^ increased. Results indicated that both -OH, C=O, C-O, aromatic structures and PO_4_^3−^ are involved in the adsorption of RhB.

#### 2.2.3. SEM and EDS Characterization

**Figure 13:**
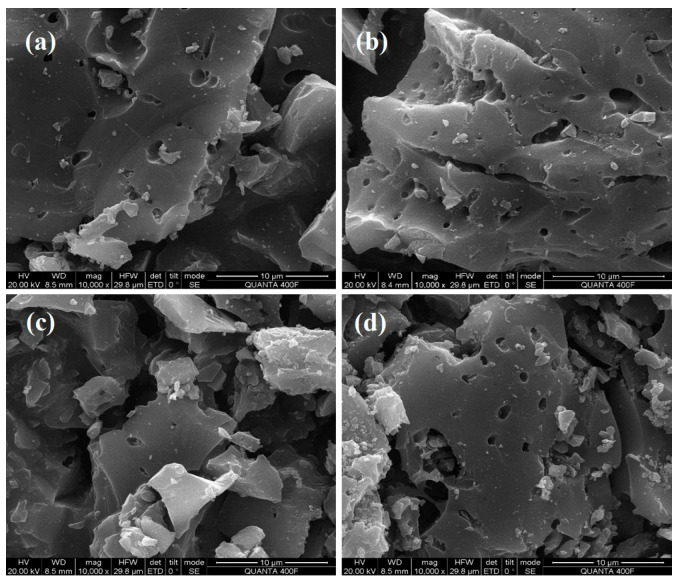
SEM spectra of PCSC; untreated-PCSC (**a**); PCSC (**b**); used-untreated PCSC (**c**); used -PCSC (**d**).

**Figure 14:**
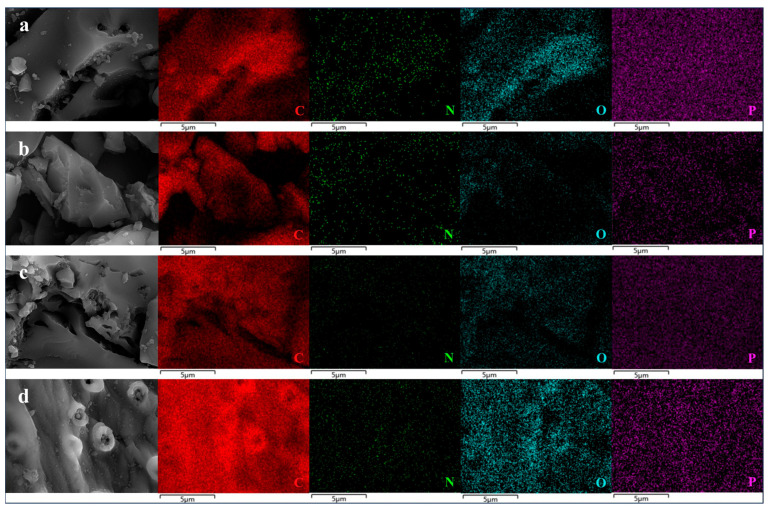
EDS spectra of PCSC; PCSC (**a**); untreated-PCSC (**b**); used -PCSC (**c**); used-untreated PCSC (**d**).

SEM as well as energy EDS and elemental mapping were employed to investigate the morphology and element distribution PCSC of untreated and used PCSC in RhB removal, which are shown in Figure 13 and Figure 14. The untreated PCSC in Figure 13a has much fewer pores than that in the treated PCSC shown in Figure 13b, and the pore size is also smaller than that of H_3_PO_4_ treated PCSC. The pore size is 0.6 μm, which is much smaller than that of PCSC, and is consistent with the characterization results of liquid UV, indicating that the modified PCSC has more small holes and that the adsorption effect of RhB is much better. Figure 13b is the SEM of PCSC. It can be seen from the figure that PCSC activated by phosphoric acid formed a large number of pores, with a pore size of 1.1 μm. There are some bumps on the surface of the carbon, which may be caused by the expansion of phosphoric acid that did not penetrate the coconut shell. Besides the bumps, there are also some shallow holes, which may be caused by the corrosion of phosphoric acid. Figure 13c shows SEM of untreated carbon after adsorption. It can be seen from the figure that the pores of PCSC are reduced, but the extent of reduction is not as high as that after adsorption of PCSC in Figure 13d, indicating that the RhB adsorbed by PCSC is not as much as that of PCSC. As can be seen from Figure 13d, the pores of PCSC are significantly reduced, but some pores still exist, indicating that the optimal PCSC adsorbs certain RhB, but not completely. EDS elemental analysis data (shown in Table 3) and mapping confirms the presence of C, O, and P elements in the PCSC and used PCSC (shown in Figure 14). The untreated PCSC also obtain C and O elements, and P less so. The P content in the modified PCSC is much higher than that in the unmodified PCSC, confirming the successful preparation of H_3_PO_4_ modified coconut shell charcoal. No N element was detected for used PCSC; it is speculated that the low concentration of adsorbed RhB resulted in a subtle increase in N content.

## 3. Materials and Methods

### 3.1. Materials and Reagents

101-OEBS electric blast drying oven, Volume: 42.875 L (Beijing Yongguangming Medical Instruments Co., Ltd., Beijing, China); SX-8-10D II box type resistance furnace, volume: 60 L, heating rate: 15 °C/min (Tianjin Test Instruments Co., Ltd., Tianjin, China); 300 W ultraviolet high pressure mercury lamp ballast (Shanghai Yaming Lighting Factory Co., Ltd., Shanghai, China); WFJ-7200 visible light spectrophotometer (Unico Shanghai Instruments Co., Ltd., Shanghai, China); BRUKER TENSOR27FT-IR Fourier transform infrared spectrometer (Shanghai Precision Scientific Instruments Co., Ltd., Shanghai, China); Y-2000x-ray diffractometer (Liaoning Dandong Aolong Instrument Co., Ltd., Dandong, China); S-4800 scanning electron microscope (Hitachi Instruments Co., Ltd., Dalian, China); Quantachrome EVO fully automatic surface area and pore size analyze (Quantachrome Instruments, Inc., Shanghai, China); ZEISS Sigma 300 scanning electron microscope (ZEISS, Inc., Oberkochen, Germany); JB-1A magnetic agitator (Hangzhou Instrument Motor Co., Ltd., Hangzhou, China); AL204 electronic balance (Shanghai Mettler Toledo Instrument Co., Ltd., Shanghai, China).

### 3.2. PSPC Sample Preparation

Principal chemical reagent: RhB (AR) (Shanghai Macklin Biochemical Technology Co., Ltd., Shanghai, China); phosphoric acid (AR) concentration: 85%, density: 1.874 g/mL (Guangdong Guanghua Sci-Tech Co., Ltd., Shantou, China); deionized water prepared by ZYPURE-II-20T reverse osmosis pure water system (Sichuan Zhuoyue Water Treatment Equipment Co., Ltd., Chengdu, China); coconut shells purchased from the agricultural market in Panlong District, Kunming City, Yunnan Province. The coconut water and meat were removed from the coconut, the remaining coconut shell was washed with tap water 2–3 times, and then it was washed with distilled water 2–3 times. It was dried in an oven at 60 °C for 24 h. After drying, the coconut shells were ground using a pulverizer, and the resulting powder was sieved to obtain particles in the 250–450 μm range. The powder was then placed in a sealed bag for later use.

Using phosphoric acid as an activator, 10 g of dried coconut shell powder was immersed in phosphoric acid solutions of different concentrations and temperatures for 4 h with magnetic stirring. Then the solution was filtered under reduced pressure, the filter residue was placed in the oven at 60 °C for 24 h, and then roasted in the Muffle furnace at different temperatures for 2 h. The crude product obtained by roasting was neutralized by washing with deionized water, then dried in an oven at 110 °C temperatures for 24 h. After cooling, it was ground and saved for later use. The carbon obtained from the coconut shell was recorded as PCSC.

### 3.3. Adsorption Experiments

#### 3.3.1. Adsorption Experiment

Taking RhB wastewater as the research objective, the amount of coir carbon m = 0.2 g. The coconut shell carbon was added to 100 mL RhB wastewater solution with a certain concentration, and the solution was placed in contact with the target molecules by magnetic stirring. Samples were taken every 5 min, and the supernatant was taken after centrifugation for 5 min. Using deionized water as a reference, after dilution (λ_max_ = 554 nm) and determination of the absorbance value according to the standard curve to calculate the residual RhB wastewater mass concentration, the RhB wastewater adsorption rate and equilibrium adsorption capacity were calculated using Formulas (1) and (2):

(1) $$R=\frac{c_{0}-c_{t}}{c_{0}}\times 100\%$$

(2) $$q_{e}=\frac{{(c}_{0}-c_{e})\times V}{m}\times 100\%$$

In this formula, R—the adsorption rate of RhB wastewater; *c*_0_—the initial mass concentration of RhB wastewater, mg/L; *c*_t_—the mass concentration of RhB at time t after adsorption by PCSC, mg/L; *q*_e_—when the adsorption reaction reaches equilibrium, the adsorption amount of RhB by PCSC, mg/g; *c*_e_—the mass concentration of RhB after adsorption equilibrium, mg/L; V—the initial volume of RhB solution, L; m—dosage of PCSC, g.

#### 3.3.2. Study on the Influence Factors of RhB Adsorption over PSPC

Calcined temperature of PCSC: H_3_PO_4_-activated coconut shell powder, calcined at 300–700 °C for 2 h, was used to obtain coconut shell charcoal. Then, 0.2 g of coconut shell charcoal was added to 100 mL of 10 mg/L initial RhB solution for 30 min to determine the optimal roasting temperature for subsequent experiments.

Initial pH values: the initial pH values of the solution were adjusted to 2–12 using 1 mol/L HCl and 1 mol/L NaOH, respectively. Then, 0.2 g PCSC was added to 100 mL 10 mg/L initial RhB solution with different pH values to investigate the effect of pH value on adsorption.

Adsorption reaction temperature: ice cubes and a constant temperature water bath were used to maintain the temperature of 100 mL of 10 mg/L RhB solution at 0, 20, 40, 60 and 80 °C, respectively. Then, 0.2 g PCSC was added to perform the adsorption reaction under stirring to investigate the effect of the reaction temperature on adsorption.

Initial concentration of RhB solution: 0.2 g PCSC was added to RhB solutions with initial concentrations of 5–50 mg/L for adsorption experiments to explore the effect of the initial concentration of crystal violet on adsorption.

The degradation effect of different pollutants: 0.2 g PCSC was added to various solutions with initial concentrations of 10 mg/L for adsorption experiments to explore the adsorption effect of coconut shell charcoal on different pollutants. The pollutants and their respective measured wavelengths are: methyl orange—464 nm; malachite green—616.9 nm; crystal violet—590 nm; bright green—422 nm; and rose red acid—478 nm. The adsorption reaction time is 30 min.

#### 3.3.3. Adsorption Ability Experiments

A total of 0.2 g of optimum coir carbon was added into RhB solution with initial concentration of 10 mg/L, and the sampling time was 5 min, 10 min, 15 min, 20 min, 25 min and 30 min. The adsorption process of optimum coir carbon on RhB over time at room temperature was explored. The optimal adsorption kinetics of RhB using coconut shell carbon was investigated by fitting the experimental data with quasi-first-order kinetics, a quasi-second-order kinetics equation and an intra-particle diffusion kinetics model.

### 3.4. Characterization of PCSC

The X-ray diffraction (XRD) patterns were obtained using a horizontal Y-2000x-ray powder diffractometer with Cu Kα (Kα = 0.15406 nm) radiation and a power of 40 kV at 30 mA. The morphology and structure of the samples were examined using a S-4800 scanning electron microscope (SEM). Fourier transform infrared (FTIR) spectra of PSPC were carried out using an FTIR instrument (BRUKER TENSOR27FT-IR, Shanghai Precision Scientific Instruments Co., Ltd., Shanghai, China). PSPC were ground with dried KBr, and the ratio of KBr to biochar particles was 200:1. In addition, the resulting mixture was pressed into pellets, and the FTIR spectra were collected at 4000–400 cm^−1^.

### 3.5. Kinetic Thermodynamic Analysis of RhB Adsorbed by PCSC

The reaction mechanism of the PCSC adsorption process can be obtained by linear fitting of quasi-first order kinetics, second-order kinetics and a particle diffusion model. The formula is as follows [29]:

(3) $$\mathrm{Quasi-first-order}\;\mathrm{kinetic}\;\mathrm{equation}:\;\ln(q_{e}-q_{t})=\ln q_{e}-k_{1}t$$

(4) $$\mathrm{pseudo-second}\;\mathrm{order}\;\mathrm{kinetic}\;\mathrm{equation}:\;\frac{t}{q_{t}}=\frac{1}{{k_{2}q_{e}}^{2}}+\frac{1}{q_{e}}t$$

(5) $$\mathrm{Particle}\;\mathrm{diffusion}\;\mathrm{model}:\;q_{t}=k_{p}t^{\frac{1}{2}}+∁$$

where *q*_t_—adsorption capacity of RhB by PCSC at time t. mg/g; *q*_e_—when the adsorption reaction reaches equilibrium, the adsorption amount of RhB by PCSC, mg/g; k_1_—quasi-first-order kinetic rate constant, min^−1^; k_2_—quasi-second-order kinetic rate constants, g/(mg·min), t—adsorption time, min; k_p_—intracelluar diffusion rate constant, mg/(g·min^1/2^); C—constant, mg/g. According to the experimental data of adsorption of RhB at different concentrations using PCSC, the *q*_t_ can be calculated by using the following Formula (6).

(6) $$q_{t}=\frac{\left(c_{0}-c_{t}\right)\times V}{m}$$

m—the dosage of PCSC, g; *c*_0_—the initial mass concentration of RhB wastewater, mg/L; *c*_t_—the mass concentration of RhB at time t after adsorption by PCSC, mg/L.

### 3.6. Thermodynamic Analysis of RhB Adsorbed by PCSC

The thermodynamic parameters of adsorption mainly include Gibbs free energy change (ΔG^θ^, kJ/mol), enthalpy change (ΔH^θ^, kJ/mol), entropy change (ΔS^θ^, J/(mol·K), and adsorption potential (E_a_, kJ/mol), which can be calculated using the following formula [30].

(7) $$\Delta G^{\theta }=-RT\ln k_{d}$$

(8) $$\ln k_{d}=\frac{\Delta S^{\theta }}{R}-\frac{\Delta H^{\theta }}{RT}$$

(9) $$E_{a}=-RT\ln\frac{c_{e}}{c_{0}}$$

In this formula, k_d_—the adsorption partition coefficient, k_d_ = *q*_e_/*c*_e_, L/mg; R—the number of gas moles, 8.314 J/(mol·K); T—the absolute temperature, K. ΔH^θ^ can be calculated using the kinetic formula.

### 3.7. Adsorption Isotherm

The adsorption isotherm is used to describe the equilibrium relationship between the adsorbent and the adsorbent, and the affinity and the adsorption capacity of the adsorbent. In the process of RhB adsorption, the adsorption isotherm can be used to determine the interaction between the adsorbent and the dye. Langmuir and Freundlich models were used to analyze the experimental data of PCSC adsorption of RhB at three different temperatures (293 K, 313 K and 333 K). The Langmuir and Freundlich equations are respectively Equations (10) and (11).

(10) $$\mathrm{Langmuir}\;\mathrm{equation}:\;q_{e}=\frac{q_{\max}\;k_{L}c_{e}}{1+k_{L}c_{e}}$$

(11) $$\mathrm{Freundlich}\;\mathrm{equation}:\;q_{e}=k_{F}{c_{e}}^{\frac{1}{n}}$$

*q*_e_—the adsorption amount of RhB by PCSC at equilibrium, mg/g; *q*_max_—maximum adsorption capacity, mg/g; k_L_—Langmuir equation adsorption constant, L/mg; *c*_e_—the mass concentration of RhB after adsorption equilibrium, mg/L; k_F_ and n—Freundlich model adsorption constants.

## 4. Kinetic, Thermodynamic Analysis and Mechanism of RhB Adsorbed on PCSC

### 4.1. Kinetic

**Figure 15:**
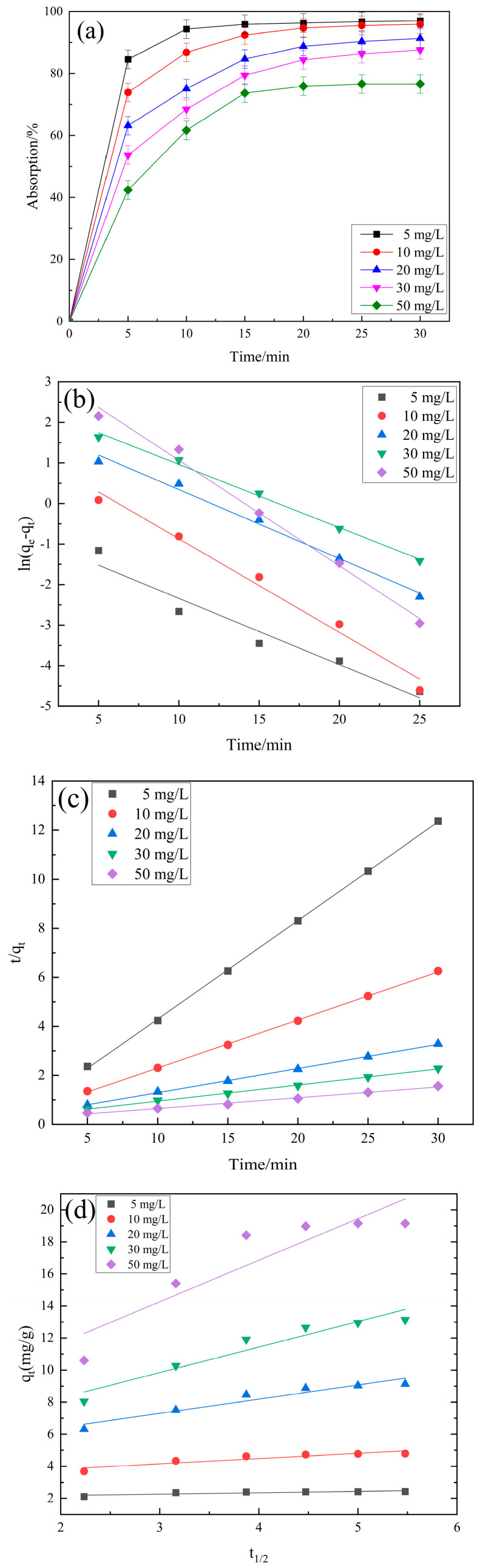
(**a**) Adsorption kinetics curves of different initial concentrations of RhB onto PCSC. (**b**) Pseudo-first-order kinetic. (**c**) Pseudo-second-order kinetic. (**d**) Intra particle diffusion model.

To study the effect of initial RhB concentration on RhB adsorption on PCSC, a fixed PCSC dosage of 2.0 g/L was treated with RhB solutions with concentrations ranging between 5 and 50 mg/L under optimum conditions; see Figure 15a. The amount of RhB adsorbed on the PCSC shows a nonlinear increase. Increasing RhB concentration increases the capacity of the fixed amount of adsorbent. As can be seen in Figure 15a, the RhB adsorption efficiency on PCSC decreases with the increase of RhB concentration. When the concentration of RhB was 5 mg/L, the adsorption rate was 97.01%. When the concentration of RhB was 50 mg/L, the adsorption rate was 76.58%, but the absorbent amount is increased with initial RhB concentration.

It can be seen from Figure 15b–d that the quasi-second-order kinetic curve is superior to other linear fits, and the fitting curve of the intraparticle diffusion model was fitted for absorption model study. And the kinetic parameters are showed in Table 4. The adsorption process is divided into two stages, corresponding to liquid film diffusion and intraparticle diffusion. In the first stage, RhB will rapidly diffuse towards PCSC; in the second stage, as the adsorption amount increases, the resistance to diffusion within the particles increases, and the speed of RhB diffusion from the particle surface to the interior slows down. Therefore, the second stage is the actual speed control step. The fitting lines of both stages did not pass through the origin, indicating that intraparticle diffusion is not the only rate controlling step. Therefore, the process of PCSC adsorbing RhB is jointly controlled by liquid film diffusion and intraparticle diffusion. The second order kinetics were more suitable to describe the adsorption process of RhB by PCSC.

### 4.2. Thermodynamic Analysis

**Figure 16:**
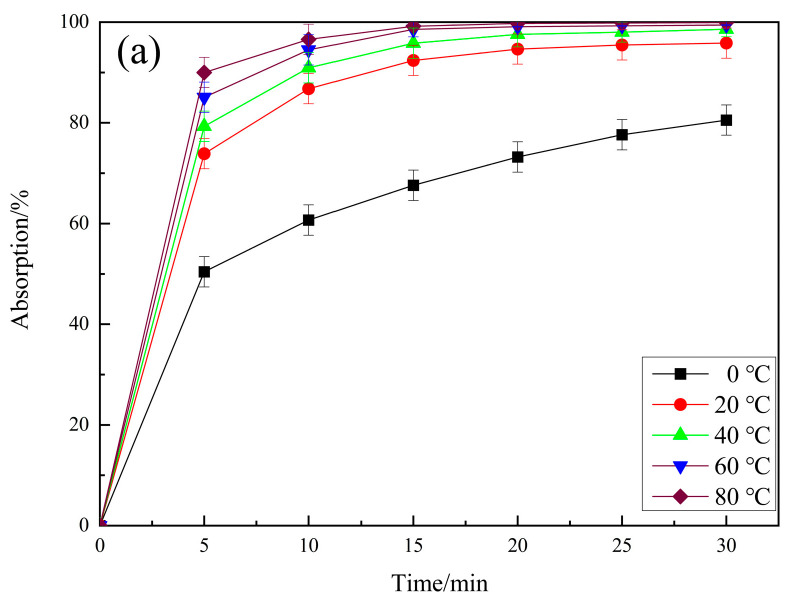

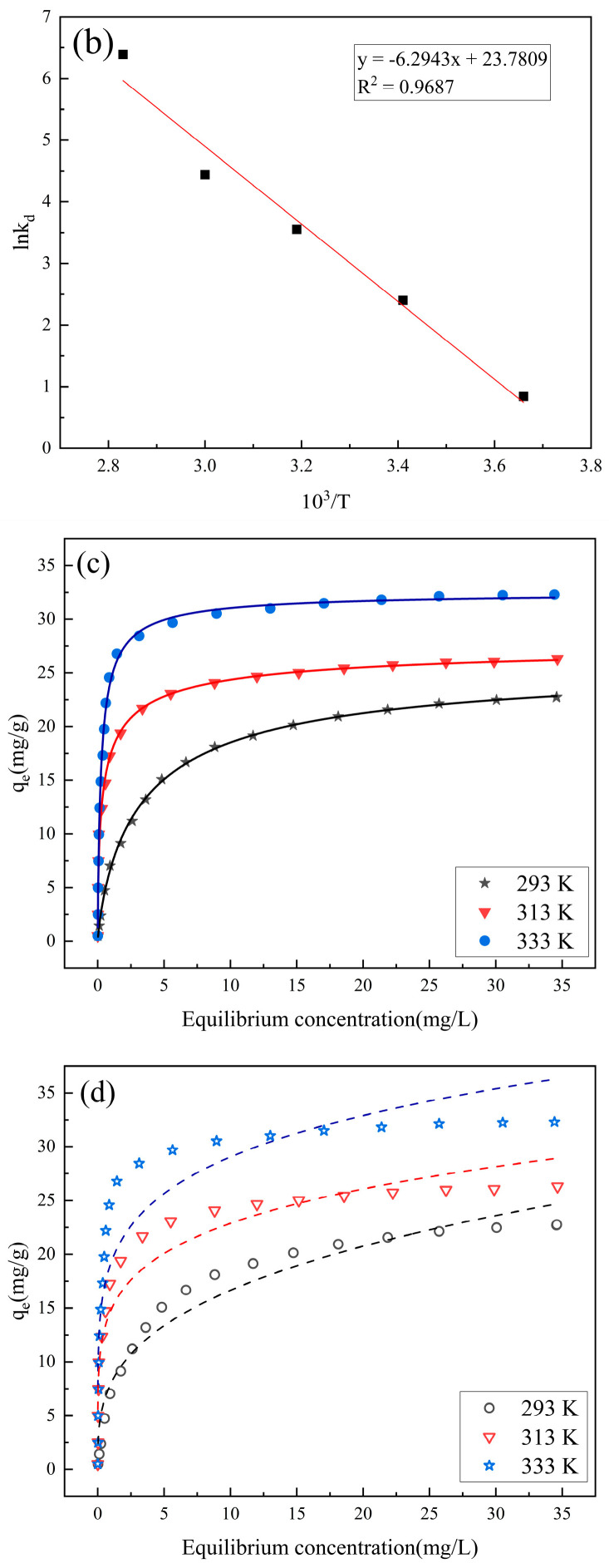
(**a**) The adsorption activity of PCSC for RhB at different temperatures. (**b**) Thermodynamic curve. (**c**) Langmuir adsorption isotherm of PCSC for RhB. (**d**) Freundlich adsorption isotherm of PCSC for RhB.

It can be seen from Figure 16a,b that with the increase of adsorption temperature, the adsorption rate of RhB by PCSC becomes faster and the adsorption rate increases. The adsorption rates of 0 °C, 20 °C, 40 °C, 60 °C and 80 °C are 80.54%, 95.84%, 98.59%, 99.41% and 99.91%, respectively. The higher the temperature, the faster the adsorption rate. At 20 °C, the adsorption rate reached 95.84% in 30 min, and 98.01% in 25 min at 40 °C. At 60 °C, the adsorption rate reached 98.56% in 15 min, while at 80 °C, the adsorption rate reached 96.57% in 10 min. The reason may be that the diffusion speed of molecules and the punching effect of water vapor on PCSC with the increase in temperature are accelerated, so that the pore size of PCSC is expanded. This is conducive to the adsorption of RhB, so the adsorption time is reduced [31].

ΔG^θ^, ΔH^θ^ and E_a_ calculated according to the thermodynamic fitting curve and formula are listed in Table 5.

Thermodynamic parameters in Table 5 were calculated using the thermodynamic curve and thermodynamic formula. ΔG^θ^ was between −1.6507 and −18.7505 kJ/mol. When the ΔG^θ^ value is between 0–20, it belongs to physical adsorption, so the adsorption of Rhodamine B by PCSC is physical adsorption.

The Langmuir and Freundlich models were applied to analyze the experimental data of PCSC adsorption of RhB at three different temperatures (293 K, 313 K, and 333 K), as shown in Figure 16c,d. In the Freundlich model, 1/n represents the degree of deviation from linearity. When 1/n < 1, it indicates easy adsorption, and when 1/n > 1, it indicates difficult adsorption [32]. The fitting data from Figure 16c,d and Table 6 indicate that its R^2^ ranges from 0.856 to 0.953, and PCSC adsorbs RhB 1/n < 1 at three different temperatures, indicating that this process is easy. The R^2^ values of the Langmuir equation are all greater than 0.991, and larger than the R^2^ values of the Freundlich equation, indicating that the Langmuir model can better describe the process of PCSC adsorption of RhB; that is, PCSC adsorption of RhB is an ideal monolayer adsorption, with a maximum monolayer adsorption capacity of 32.57 mg/g at 333 K [33].

### 4.3. Adsorption Mechanism

**Figure 17:**
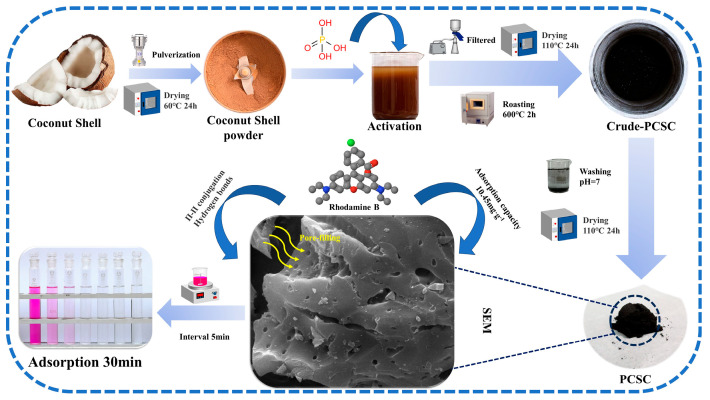
The possible mechanism of RhB adsorption on PCSC.

A possible reaction mechanism was proposed using RhB adsorption data of PCSC under different conditions and combining with structure characterization. H_3_PO_4_ activated PCSC has a higher specific surface area and microporous structure. More oxygen-containing functional groups and PO_4_^3−^ could enhance the adsorption capacity of RhB. Through the influence of pH value on adsorption performance, it is shown that PCSC is prone to forming hydrogen bonds with RhB. According to the UV-VIS spectrum, the characteristic peaks do not shift during the adsorption of RhB by activated PCSC, indicating that adsorption is mainly physical adsorption. Based on the FTIR spectra, the changes in functional groups before and after the PCSC adsorption of RhB indicate that the process of PCSC adsorption of RhB includes pore filling, π−π conjugation between the RhB conjugated system and the benzene ring structure of PCSC, and hydrogen bonding and electrostatic forces. The schematic diagram of the reaction mechanism is shown in Figure 17.

## 5. Conclusions

(1) Using coconut shell as the material and H_3_PO_4_ as the modifier reagent, PCSC was prepared. For RhB adsorption, 2 mol/L H_3_PO_4_ impregnation for 4 h under 40 °C water bath and calcined at 600 °C for 2 h has the best removal efficiency. When the optimal adsorption acidity was pH = 6, the adsorption rate reached 95.84%. After 6 cycles, the absorption efficiency of PCSC can still be maintained above 85%. Also, the obtained PCSC exhibits excellent adsorption performance for the other five common dyes.

(2) The characterization of PCSC showed that the functional groups indicated on PCSC decreased gradually with calcined temperature, and the degree of carbonization increased continuously. The optimal PCSC treated with phosphoric acid formed a larger number of micropores than untreated PCSC. After adsorption, the number of micropores decreased on the optimal PCSC. Kinetic and thermodynamic analyses show that the quasi-second-order kinetics are more suitable to describe the adsorption process of RhB by PCSC, and the adsorption is a spontaneous endothermic reaction. The obtained PCSC sorption isotherms were classified as Langmuir-type.

(3) Combined with SEM and FTIR, PCSC adsorption of RhB mechanism includes pore diffusion, hydrogen bonding, and π−π conjugation. The well-developed pore structure of PCSC facilitates the physical diffusion of RhB molecules. The abundant hydroxyl and ester groups, aromatic structures, and PO_4_^3−^ on the surface of PCSC can form hydrogen bonds and π−π conjugation, and the higher surface area and micropore distribution promoted the PCSC adsorption RhB efficiency.

## Figures and Tables

**Table 1:** Relationship between dye properties and adsorption rates.

Dye	Structure Fomula	Molecular Weight	Dye Type	Removal Efficiency/%
RhB	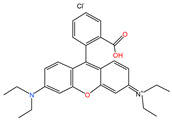	479.01	Cationic dye	95.84
Methyl orange	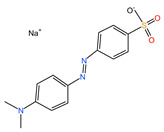	327.33	Anionic type	76.55
Malachite green	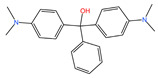	364.92	Cationic dye	91.79
Bright Green	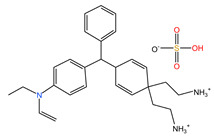	482.63	Cationic dye	46.26
Rose Red Acid	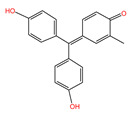	304.34	Anionic type	73.92
Crystal Violet	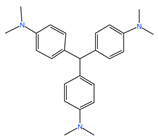	373.53	Cationic dye	94.59

**Table 2:** Comparison with other adsorbents.

Adsorbent	pH	Adsorbent Dosage (g L^−1^)	q_e_ (mg g^−1^)	Ref
LCNFS	2–7	/	20.2	Zhang et al., 2016 [22]
LPC@La(OH)_3_	3–7	1.25	31.9	Liu et al., 2019 [23]
ML2-CGCS	3–8	1.4	39.2	Wang et al., 2016 [24]
CB	10	5	96.5	Oladipo, Akeem Adeyemi, 2017 [25]
PCSC	6	2	32.6	This work

**Table 3:** EDS elemental analysis data of different samples.

Sample and Elements Content (wt.%)	C	N	O	P
PCSC(a)	88.14	0	7.95	3.91
Untreated-PCSC(b)	94.19	0	5.46	0.36
Used -PCSC(c)	88.50	0	8.19	3.30
Used-untreated PCSC(d)	94.83	0	5.17	0

**Table 4:** Kinetic parameters.

Initial Concentration C_0_/(mg/L)	q_e_/(mg/g)	Quasi-First-Order Dynamics		Quasi-Second-Order Dynamics		Particle Diffusion Model		
		K_1_	R_1_^2^	K_2_	R_2_^2^	K_p_	C	R^2^
5	2.4277	0.1286	0.9959	0.4020	0.9998	0.0845	2.0118	0.6499
10	4.7832	0.1692	0.9609	0.1961	0.9996	0.3262	3.1746	0.8141
20	9.1329	0.0873	0.9701	0.0986	0.9992	0.8846	4.6505	0.9067
30	13.1936	0.0892	0.9446	0.0657	0.9987	1.5892	5.0805	0.9071
50	19.2052	0.1257	0.9655	0.0439	0.9891	2.5889	6.4994	0.7829

**Table 5:** Thermodynamic parameters.

C/(mg/L)	ΔH^θ^/(kJ/mol)	ΔS^θ^J/(mol·K)	ΔG^θ^/(kJ/mol)					E_a_/(kJ/mol)				
			273 K	293 K	313 K	333 K	353 K	273 K	393 K	313 K	333 K	353 K
10	52.3312	197.9109	−1.6507	−5.8486	−9.2411	−12.2973	−18.7505	3.7151	7.6451	11.0898	14.2325	20.7872

**Table 6:** Parameters of the adsorption isotherm equation for RhB on PCSC.

Temperature T/(K)	Langmuir Equation			Freundlich Equation		
	q_max_/(mg/g)	k_L_/(L/mg)	R^2^	k_F_/(L/mg)	1/n	R^2^
293	26.3045	0.3570	0.9993	8.0156	0.3174	0.9537
313	28.1741	1.6468	0.9917	14.8143	0.1887	0.9193
333	32.5769	2.9828	0.9967	19.2044	0.1797	0.8569

## Data Availability

The original contributions presented in the study are included in the article.
